# Enterogenous bacterial glycolipids are required for the generation of natural killer T cells mediated liver injury

**DOI:** 10.1038/srep36365

**Published:** 2016-11-08

**Authors:** Yingfeng Wei, Benhua Zeng, Jianing Chen, Guangying Cui, Chong Lu, Wei Wu, Jiezuan Yang, Hong Wei, Rufeng Xue, Li Bai, Zhi Chen, Lanjuan Li, Kazuya Iwabuchi, Toshimitsu Uede, Luc Van Kaer, Hongyan Diao

**Affiliations:** 1 State Key Laboratory for Diagnosis and Treatment of Infectious Diseases, Collaborative Innovation Center for Diagnosis and Treatment of Infectious Diseases, The First Affiliated Hospital, College of Medicine, Zhejiang University, Hangzhou, Zhejiang, 310003, China; 2 Department of Laboratory Animal Science, College of Basic Medical Sciences, Third Military Medical University, Chongqing, 400038, China; 3 Institute of Immunology and Key Laboratory of Innate Immunity and Chronic Disease of CAS, School of Life Sciences and Medical Center, University of Science and Technology of China, Hefei 230027, China; 4 Innovation Center for Cell Biology, Hefei National Laboratory for Physical Sciences at Microscale, Hefei 230027, China; 5 Department of Immunology, Kitasato University School of Medicine, Sagamihar, 108-8641, Japan; 6 Molecular Immunology, Institute for Genetic Medicine, Hokkaido University, Sapporo, 0600815, Japan; 7 Department of Microbiology and Immunology, Vanderbilt University School of Medicine, Nashville, Tennessee, 37232, USA.

## Abstract

Glycolipids are potent activator of natural killer T (NKT) cells. The relationship between NKT cells and intestinal bacterial glycolipids in liver disorders remained unclear. We found that, in sharp contrast to specific pathogen-free (SPF) mice, germ-free (GF) mice are resistant to Concanavalin A (ConA)-induced liver injury. ConA treatment failed to trigger the activation of hepatic NKT cells in GF mice. These defects correlated with the sharply reduced levels of CD1d-presented glycolipid antigens in ConA-treated GF mice compared with SPF counterparts. Nevertheless, CD1d expression was similar between these two kinds of mice. The absence of intestinal bacteria did not affect the incidence of αGalCer-induced liver injury in GF mice. Importantly, we found the intestinal bacteria contain glycolipids which can be presented by CD1d and recognized by NKT cells. Furthermore, supplement of killed intestinal bacteria was able to restore ConA-mediated NKT cell activation and liver injury in GF mice. Our results suggest that glycolipid antigens derived from intestinal commensal bacteria are important hepatic NKT cell agonist and these antigens are required for the activation of NKT cells during ConA-induced liver injury. These finding provide a mechanistic explanation for the capacity of intestinal microflora to control liver inflammation.

Natural killer T (NKT) cells are unconventional T cells that express both T cell receptors (TCRs) and natural killer (NK) cell receptors. NKT cells are predominantly express an invariant TCRα-chain formed by α-chain variable region 14-α-chain joining region 18 (Vα14-Jα18) rearrangement in mice and Vα24-Jα18 rearrangement in humans^1^. Unlike conventional T cells, NKT cells recognize glycolipid antigens that are presented by the major histocompatibility complex class I-like molecule CD1d^2^. CD1d presented glycolipids could subsequently cause the activation of NKT cells.

The liver harbors many NKT cells, which are closely linked to liver dysfunction, such as hepatitis and hepatocellular carcinoma^3^,^4^. Concanavalin A (ConA)-induced hepatitis is a widely used mouse model for studying liver-associated diseases. Studies have shown that the activation of hepatic NKT cells play a central role in ConA-induced liver injury, both CD1d- and Jα18-deficient mice that lack of NKT cells are resistant to ConA-induced liver injury^5^,^6^. After activation, NKT cells upregulated their activation marker and rapidly secrete a variety of cytokines, including IFN-γ and IL-4. NKT cells can directly cause liver injury by Fas/Fas ligand (FasL) mechanism and they secrete various cytokines that recruit and activate other innate immune cells to exacerbate inflammatory responses in the liver^6^. Besides, administration of α-galactosylceramide (αGalCer), a typical glycolipid antigens derived from marine sponges, leads to rapid activation of hepatic NKT cells and causes significant liver injury in mice^7^. This indicated that NKT-recognized glycolipids could induce NKT-mediated liver injury *in vivo*; however, whether the enterogenous glycolipid antigens could serve as the activator of NKT cell in the liver injury process remains elusive.

Increasing attention is paid to the study of the relationship between liver diseases and intestinal microbiota. The human intestine is populated by a large variety of microorganisms that colonize the host soon after birth, the gut microflora are closely linked to host physiology and pathology^8^. In particular, gut microflora and their products contribute to a variety of liver disorders^9^,^10^. Glycolipid antigens show a widespread distribution in bacteria, but just a small number of bacteria were found containing the NKT recognized glycolipids. Recent studies suggested that glycolipids derived from *Sphingomonas*, *Borrelia burgdorferi*, *Streptococcus pneumoniae* and group B *Streptococcus* are recognized by NKT cells^1^,^2^. However, whether the intestinal commensal bacteria contain NKT recognized glycolipids is still not very clear. Although, the involvement of intestinal bacteria or hepatic NKT cells in liver disorders has been firmly established, respectively, the relationship between intestinal bacteria-derived glycolipids and hepatic NKT cells in liver injury remains unclear.

We found that, in contrast to specific pathogen-free (SPF) mice, germ-free (GF) mice were resistant to ConA-induced liver injury and NKT cell activation. Importantly, the quantity of CD1d-presented glycolipid antigens after ConA treatment was significantly higher in SPF mice compared to GF mice. Result revealed that enterogenous bacterial glycolipids are important NKT cell activator and are required for activation of hepatic NKT cells during liver injury. These finding provide a mechanistic explanation for the capacity of intestinal microflora to control liver inflammation.

## Results

### GF mice are resistant to ConA-induced liver injury

**Figure 1:**
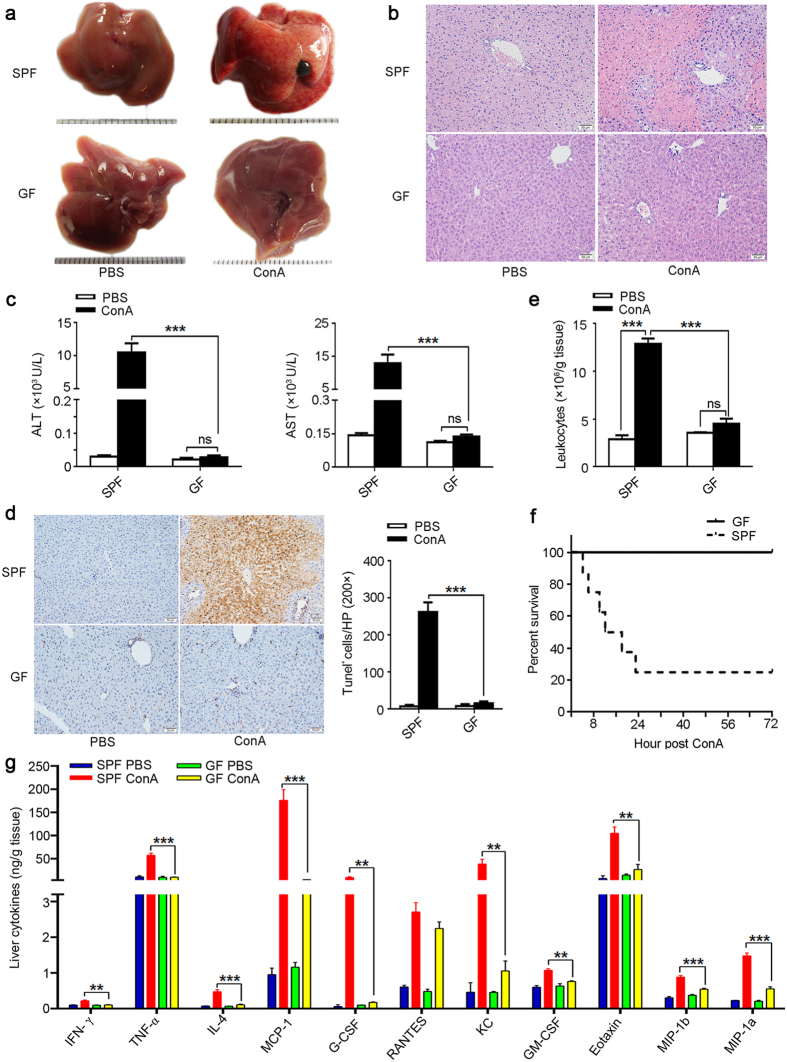
GF mice fail to develop ConA-induced liver injury. **(a–e**,**g)** ConA or PBS was injected into SPF or GF BALB/c mice, after 24 hr mice were sacrificed. (**a**) Gross appearances of livers. Scale bar represents 0.1 cm. (**b**) Representative histological appearance (H&E staining) of livers. Original magnification, 200×, scale bar represents 50 μm. (**c**) Serum ALT and AST levels. (**d**) TUNEL assay of liver sections. Original magnification, 200×, scale bar represents 50 μm. The TUNEL-positive cells per field are shown in the right panel (calculated by Image J software). (**e**) The number of intrahepatic infiltrating leukocytes. (**f**) Survival analysis of mice after injecting a lethal dose of ConA (30 mg/kg), survival was monitored for 72 hr and log-rank test was used to assess for differences in survival. (**g**) Cytokine profile of the liver was analyzed by the Luminex enzyme immunoassay system. The data represent means ± SEM (n = 8, two independent experiments). **P < 0.01, ***P < 0.001, ns represents not significant, (**c**–**d**,**g**) One-way ANOVA.

To investigate the contribution of the intestinal microflora to the pathogenesis of liver injury, we injected ConA into GF and SPF mice. We found severe liver damage in SPF mice after ConA challenge, as reflected by gross liver appearance (Fig. 1a), liver H&E staining (Fig. 1b), and serum ALT and AST levels (Fig. 1c). Interestingly, we found GF mice were resistant to ConA-induced liver injury (Fig. 1a–c). To further characterize the degree of liver damage, we measured apoptosis in tissue sections. In contrast to SPF mice, apoptosis was nearly undetectable in the liver of ConA-treated GF mice (Fig. 1d). In addition, we assessed the numbers of liver-infiltrating leukocytes, which reflects ongoing levels of liver inflammation, found that leukocyte infiltration was significantly lower in ConA-treated GF mice compared to SPF mice (Fig. 1e). Importantly, survival was substantially increased in GF mice. Three days after ConA treatment most SPF mice had died, whereas all GF mice were still alive (Fig. 1f). We also found that the levels of inflammatory cytokines, including IFN-γ, TNF-α, IL-4, MCP-1, G-CSF, KC, GM-CSF, Eotaxin, MIP-1b and MIP-1a were significantly higher in the liver of ConA-treated SPF mice than GF mice (Fig. 1g). Profile of these cytokines in the serum was largely similar to the liver (Supplementary Fig. S1). These data provide strong evidence for GF mice resistant to ConA-induced hepatic injury.

### ConA treatment fails to activate hepatic NKT cells from GF mice

**Figure 2:**
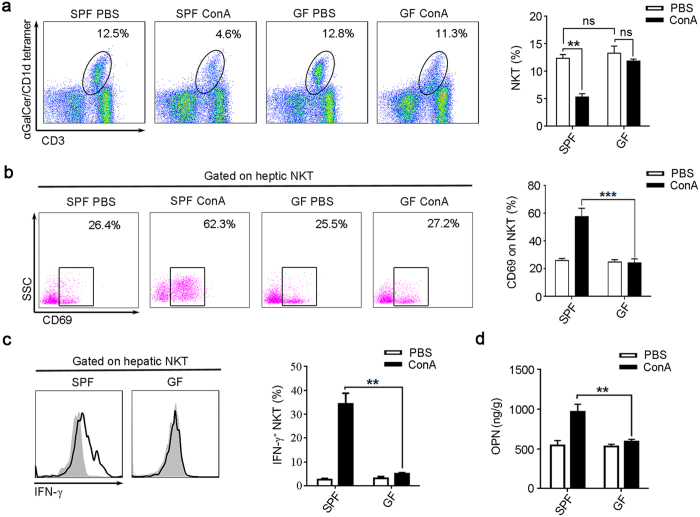
ConA is unable to activate NKT cells from GF mice. BALB/c mice were sacrificed 24 hr after ConA or PBS injection. (**a**–**c**) Intrahepatic leukocyte was analyzed by flow cytometry. (**a**) The percentages of NKT cells (CD19^−^CD3^+^αGalCer/CD1d tetramer^+^) were determined, the representative scatterplot (left panel) and summative histogram (right panel) are shown. (**b**) Measurements of CD69 expression on hepatic NKT cells. (**c**) The intracellular IFN-γ levels in hepatic NKT cells, the filled flow cytometric histograms represent PBS-treated mice, and the open histograms represent ConA-treated mice. (**d**) OPN levels in the liver tissue, analyzed by Elisa. Two independent experiments with similar results were performed. The data represent means ± SEM (n = 8, two independent experiments). **P < 0.01, ***P < 0.001, ns represents not significant, One-way ANOVA.

Previous studies have established that NKT cell has a pivotal role in the induction of mice liver injury by ConA^5^,^11^. Activation of NKT cells result in a rapid decline in the frequency of hepatic NKT cells, due to profound downregulation of NKT cell receptors^6^,^11^,^12^,^13^. Consistent with these studies, we found that the percentages of NKT cells were sharply reduced in the liver of ConA-treated SPF mice (Fig. 2a). But, we did not detect significant down modulation of NKT cells in ConA-treated GF mice (Fig. 2a). In addition, we found ConA treatment increased the CD69 expression in hepatic NKT cells of SPF mice, which was not observed in GF mice (Fig. 2b). Previous study reported that activated NKT cells express higher IFN-γ^14^. We found the IFN-γ production of NKT cell was significantly higher in ConA-treated SPF mice than GF mice (Fig. 2c). Our previously study also found that activated NKT cell could secrete more osteopontin (OPN)^5^, OPN augments the neutrophil infiltration and activation^5^,^11^,^15^. In current study, we found the expression of hepatic OPN were significantly increased in ConA treated SPF mice compared to GF mice (Fig. 2d). We also detected increased infiltration and activation of neutrophil cells in the liver of ConA treated SPF mice, but was not observed in the GF counterparts (Supplementary Fig. S2). These results confirmed that ConA failed to activate NKT cells in GF mice.

Additionally, we found that the frequencies of hepatic conventional T cells and NK cells, as well as the surface CD69 of conventional T cells were similar between SPF and GF mice (Supplementary Fig. S3a–c). Besides, the absolute numbers of hepatic T cell and NK cell were significantly increased after ConA injection in the SPF mice, but remain unchanged in the GF mice (Supplementary Fig. S3d–e). ConA treatment did not significantly change the absolute number of hepatic NKT cell in both SPF and GF mice (Supplementary Fig. S3f). We speculated that T and NK cells were not recruited to the liver of GF mice after ConA administration due to GF mice fail to develop ConA-induced liver injury.

### SPF and GF mice exhibit similar responses to αGalCer-induced liver damage

**Figure 3:**
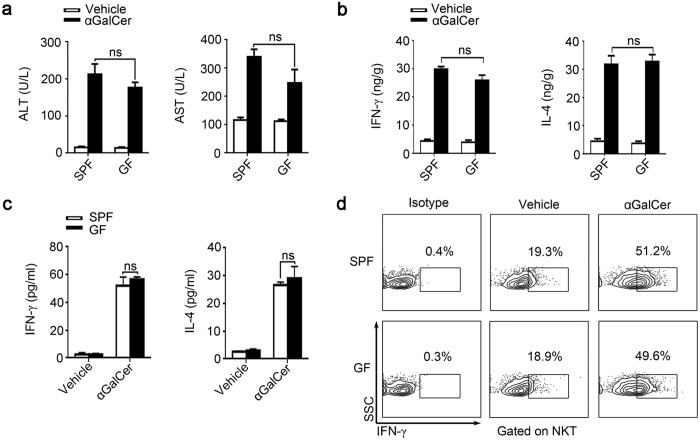
NKT cells in SPF and GF mice have similar function. (**a**,**b**) αGalCer (250 μg/kg) or vehicle were injected into BALB/c mice, samples were analyzed 24 hr post-injection. (**a**) Serum ALT and AST levels. (**b**) Liver IFN-γ and IL-4 levels were measured by ELISA. (**c**,**d**) Splenic cells from untreated SPF and GF mice were cultured with the stimulation of either αGalCer or vehicle for 24 hr. (**c**) IFN-γ and IL-4 levels in the supernatant were analyzed by Elisa. (**d**) Intracellular IFN-γ expression in NKT cells were analyzed by flow cytometry. The data represent means ± SEM (n = 8), ns represents not significant, One-way ANOVA.

Report have demonstrated that injection of αGalCer, a specific activator for NKT cells, also can induce liver injury^7^. Upon injection of αGalCer into SPF and GF mice, we found the ALT and AST levels were elevated to similar levels in both groups of animals (Fig. 3a). The IFN-γ and IL-4 levels in GF and SPF mice were also similar, regardless of treatment (vehicle or αGalCer) (Fig. 3b). Similar results were acquired in the *in vitro* experiment that cultured the splenic cells from untreated SPF and GF mice. Irrespective of αGalCer stimulation, we found that SPF and GF mice have parallel IFN-γ and IL-4 levels in culture supernatant (Fig. 3c), more importantly, the intracellular IFN-γ of NKT cells was also similar between these two kinds of mice (Fig. 3d). These data show that αGalCer-induced liver injury in SPF and GF mice is comparable, and the functions of NKT cells in GF and SPF mice are similar.

### APCs from SPF and GF mice have similar phenotypes and CD1d expression

**Figure 4:**
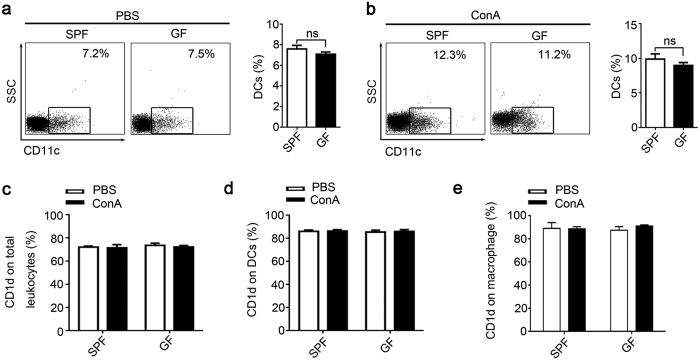
Analyze of APCs phenotypes and CD1d expression. Liver infiltrating leukocytes were prepared from SPF and GF BALB/c mice 24 hr after ConA or PBS injection. The percentages of CD11c^+^ DCs after (**a**) PBS or (**b**) ConA treatment were determined by flow cytometry. The CD1d expression of (**c**) total intrahepatic leukocytes, (**d**) DCs (CD11c^+^) and (**e**) macrophage cells. The data represent means ± SEM (n = 8, two independent experiments), ns represents not significant, (**a**,**b**) Mann-Whitney U test, (**c–e**) One-way ANOVA.

NKT cells recognize glycolipids that are presented by CD1d. Antigen-presenting cells (APCs) such as dendritic cells (DCs) and macrophages express high levels of CD1d molecules, and study showed DCs were most effectively in presenting αGalCer analogs glycolipids^16^. We found that the percentages of intrahepatic DCs were not different between PBS-treated GF and SPF mice (Fig. 4a). After ConA injection hepatic DCs were increased in SPF and GF mice with a parallel extent (Fig. 4b). Similar results were obtained by immunohistochemical staining of hepatic CD11c^+^ cells (Supplementary Fig. S4a). We further found that the CD1d expression, between PBS- or ConA-treated GF and SPF mice, on total intrahepatic leukocytes (Fig. 4c), hepatic DCs (Fig. 4d and Supplementary Fig. S4b) and macrophages (Fig. 4e) were analogous, respectively. Similar results were obtained from the spleen (Supplementary Fig. S4c,d). Additionally, the levels of MHC-II, CD80, and CD86 on hepatic DCs (Supplementary Fig. S4e,f) between GF and SPF mice also showed no differences. Collectively, these data show that the APCs phenotypes and CD1d expression are similar between SPF and GF mice, and ConA treatment does not affect CD1d expression levels in these animals.

### ConA-treated GF mice have lower glycolipid levels

**Figure 5:**
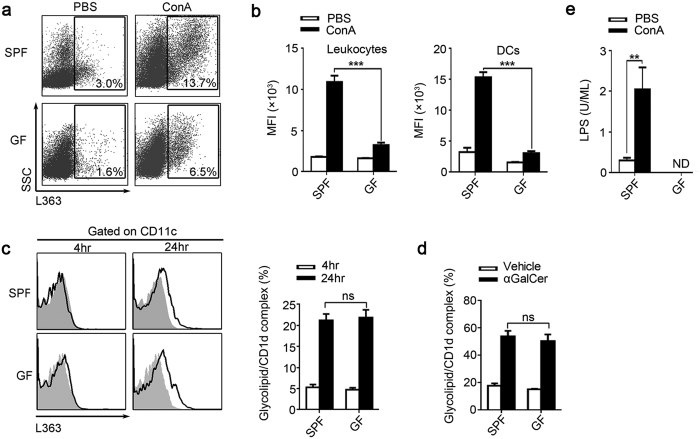
Levels of CD1d-presented and un-presented glycolipids are substantially lower in GF mice. (**a**,**b**) SPF and GF BALB/c mice were sacrificed 24 hr after ConA or PBS injection. (**a**) Using mAb L363, we detected the percentage of glycolipid/CD1d complexes in the intrahepatic leukocytes, gated on total leukocytes. (**b**) The mean fluorescence intensity (MFI) of glycolipid/CD1d complexes on total intrahepatic leukocytes and DCs. (**c**) To compared the glycolipids presenting ability between SPF and GF mice, splenic cells from untreated SPF and GF BALB/c mice were cultured with either αGalCer or vehicle stimulation for 4 and 24 hr. Then used L363 to analysis the glycolipid/CD1d complex expression on DCs by flow cytometry, filled flow cytometric histograms represent cultured with vehicle, and opened histograms represent cultured with αGalCer. (**d**) Mice were sacrificed 24 hr post αGalCer (250 μg/kg) or vehicle injection. Using L363, the glycolipid/CD1d complex on hepatic DCs was analyzed by flow cytometry. (**e**) Plasma LPS levels of SPF and GF mice 24 hr after ConA or PBS injection. The data represent means ± SEM (n = 8, two independent experiments), **P < 0.01, ***P < 0.001, ns represents not significant, One-way ANOVA.

Glycolipids are potent activator of NKT cell, they activate NKT cell mainly through the antigen presentation by CD1d. Monoclonal antibody L363 only recognize glycolipid/CD1d complexes formed by loading CD1d with αGalCer or other glycolipids which were structurally similar to αGalCer, such as C20:2 and OCH, but this mAb could not recognize CD1d without loading glycolipids^16^,^17^. Employing mAb L363 to visualize and quantify the glycolipids that presented by CD1d, we found that the percentage of intrahepatic leukocytes which expressing glycolipid/CD1d complexes in SPF mice was significantly increased after ConA treatment. However, in ConA-treated GF mice, glycolipid/CD1d complexes were significantly lower compared with SPF mice (Fig. 5a). Furthermore, more pronounced differences were observed in the MFI levels of L363 staining on both total intrahepatic leukocytes and hepatic DCs (Fig. 5b). Additionally, the levels of splenic cells expressing glycolipid/CD1d complexes in GF mice were significantly lower than those in SPF mice, regardless of the treatment (PBS or ConA) the animals received (Supplementary Fig. S5). Furthermore, by measuring the glycolipid/CD1d complexes on DCs after αGalCer or vehicle treatment *in vitro* and *in vivo*, we found the CD1d-restricted glycolipid antigen-presenting capacity of DCs from SPF and GF mice was similar (Fig. 5c,d). It suggested that the lower glycolipids that presented by the CD1d in GF mice was not due to the attenuated function of CD1d.

In addition, lipopolysaccharide (LPS) is another common bacterial antigen, but the presentation of LPS could not be detected by mAb L363^14^,^17^. Thus, we evaluated the circulating LPS and found that LPS was significantly increased in the ConA-treated SPF mice but undetectable in GF mice (Fig. 5e). LPS could activate hepatic Kupffer cells, leading to liver injury in mice. To clarify the role of Kupffer cells in this study, we further analyzed the ALT level, hepatic NKT cell and the F4/80^+^ cell of the SPF mice at indicated times after ConA injection (Supplementary Fig. S6). We found that the ALT level was increased at 2 hr after ConA injection, and the ALT level was positively correlated with the time post ConA injection within 24 hr. We found that the phenomenon of NKT downregulation have emerged at 2 hr after ConA injection, suggesting that the NKT cells activate very fast after ConA injection. However, the cell percentage of hepatic F4/80^+^ cells was not significantly changed within 12 hr after ConA injection, it significantly increased at 24 hr after ConA injection. Similar to the percentage of F4/80^+^ cells, we found that the intracellular TNF-α secretion level of hepatic F4/80^+^ cell was just increased at 24 hr after ConA injection (Supplementary Fig. S6d). In summary, these data showed that the liver injury and hepatic NKT activation emerged very early after ConA injection; however, the hepatic F4/80^+^ cell was not significantly changed within 12 hr. It suggested that NKT activation play a critical role in the ConA-induced liver injury, but the Kupffer cells was less important. We speculated that the increased number and TNF-α secretion of the hepatic F4/80^+^ cell at 24 hr after ConA injection was due to the liver injury promote the recruitment and activation of Kupffer cells.

### Increased glycolipids presenting by CD1d were from intestinal bacteria

We speculated that the increased glycolipids in ConA-induced hepatitis were derived from the intestinal bacteria, and we performed experiments to confirm this speculation. We found that ConA treatment in SPF mice significantly increased the percentage of glycolipid/CD1d complex in both portal blood lymphocyte and the intrahepatic leukocytes; however, remove most of the intestinal bacteria by antibiotics prior to ConA treatment significantly decreased the percentage of glycolipid/CD1d complex in both portal blood and liver (Supplementary Fig. S7). Importantly, orally given heat-killed intestinal bacteria mixture to the antibiotics treated mice prior to ConA injection could significantly increase the percentage of glycolipid/CD1d complex in both portal blood and liver (Supplementary Fig. S7). These data indicated that the increased glycolipids were derived from the intestinal bacteria of the mice. Moreover, we found ConA treatment disrupt the intestinal barrier which also suggested the increased glycolipids were derived from the intestinal bacteria. We found that ConA treatment could cause significant edema and degradation of the intestinal villus, especially in the SPF mice (Supplementary Fig. S8a). And the levels of several cytokines, including IFN-γ, IL-4, G-CSF, RANTES, MIP-1a and IL-6, were increased in the intestine after ConA treatment. Moreover, these cytokines were significantly higher in the SPF ConA treated mice compared to the GF ConA treated mice (Supplementary Fig. S8). These data indicated that ConA treatment can disrupt the intestinal barrier and cause intestinal inflammation, and this effect was more noticeable in the SPF mice compared to the GF mice, which suggesting that ConA effect to the intestine was closely related to the severity of liver injury. To further study the influence of ConA to gut permeability, we used the FITC-labeled dextran to compare the gut permeability between ConA and PBS treated SPF mice. We found that, compared to the PBS group, ConA treatment significantly increased intestinal permeability to FITC-labeled dextran tracer (Supplementary Fig. S8c).

### Heat-killed intestinal bacteria restore ConA-induced liver injury in GF mice

**Figure 6:**
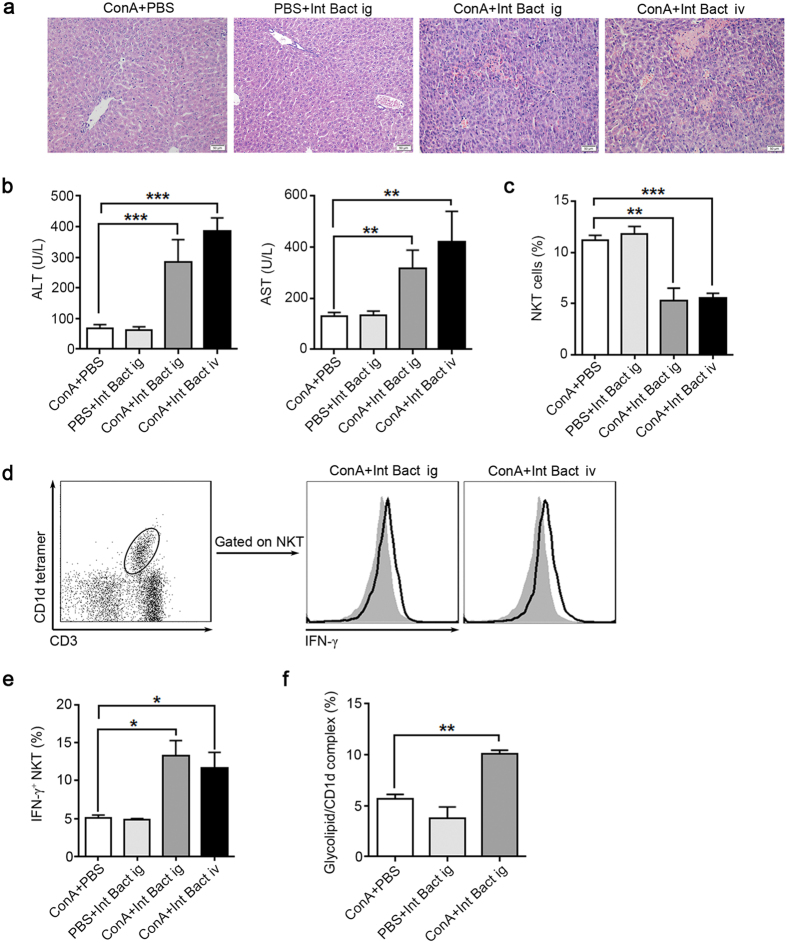
ConA-induced liver injury is restored in bacteria-treated GF mice. Except for the ‘ConA+PBS’ group, other groups were pretreated with a mixture of killed intestinal bacteria (Int Bact) prior to ConA injection by intragastric gavage (ig) or intravenous injection (iv), 8 GF BALB/c mice per group. “ConA + PBS” group received 8 day PBS orally administration followed by ConA injection, “PBS + Int Bact ig” group received 8 day Int Bact orally administration followed by PBS injection. “ConA + Int Bact ig” group received 8 day Int Bact orally administration followed by ConA injection. “ConA + Int Bact iv” group received an Int Bact injection followed by ConA injection (see details in the supplementary methods). Int Bact was consisted of *E. coli*, *E. faecalis*, *Lactobacillus*, *Salmonella enteritidis* and Group A *Streptococcus*. (**a**) H&E staining of livers. Original magnification, 200×, scale bars represents 50 μm. (**b**) Serum ALT and AST levels. (**c**) Percentages of hepatic NKT cells in intrahepatic leukocytes. (**d**) Intracellular IFN-γ levels in hepatic NKT cells, filled flow cytometric histograms represent GF mice with PBS treatment before ConA injection, and open histograms represent GF mice pretreated with Int Bact before ConA injection. (**e**) The percentages of IFN-γ^+^ NKT cells in total hepatic NKT cells. (**f**) The level of glycolipid/CD1d complex of total intrahepatic leukocytes, detected by L363 through flow cytometry. The data represent means ± SEM (n = 8, two independent experiments), *P < 0.05, **P < 0.01, ***P < 0.001, (**b**,**e**) One-way ANOVA, **(f)** Mann-Whitney U test.

Different from GF mice, SPF mice have intestinal microbiota. And we have showed that the increased glycolipids in the liver after ConA administration were derived from the intestinal. Therefore, we want to know that whether administered heat-killed intestinal bacteria mixture (Int Bact) to GF mice prior to ConA treatment could restore the ConA-induced liver injury. We found that ConA-induced liver injury was significantly enhanced in GF mice that pretreated with Int Bact compared with untreated GF mice (Fig. 6a,b). ConA administration in bacteria treated GF mice induced a decline in hepatic NKT cell (Fig. 6c), which was accompanied with increased intracellular IFN-γ levels in these cells (Fig. 6d,e). In addition, compared with control mice, the expression of glycolipid/CD1d complexes in the liver were increased in the bacteria pretreated ConA GF mice (Fig. 6f). We also found that elimination most of the intestinal bacteria by antibiotics administration ameliorated ConA-induced liver injury in SPF mice, moreover, the intracellular IFN-γ levels in hepatic NKT cells were reduced in the antibiotics treated SPF mice after ConA injection (Supplementary Fig. S9). These results suggested that intestinal bacteria are required in the ConA-induced NKT cell mediated liver injury.

### Intestinal bacteria contain glycolipids which can be recognized by NKT cells

**Figure 7:**
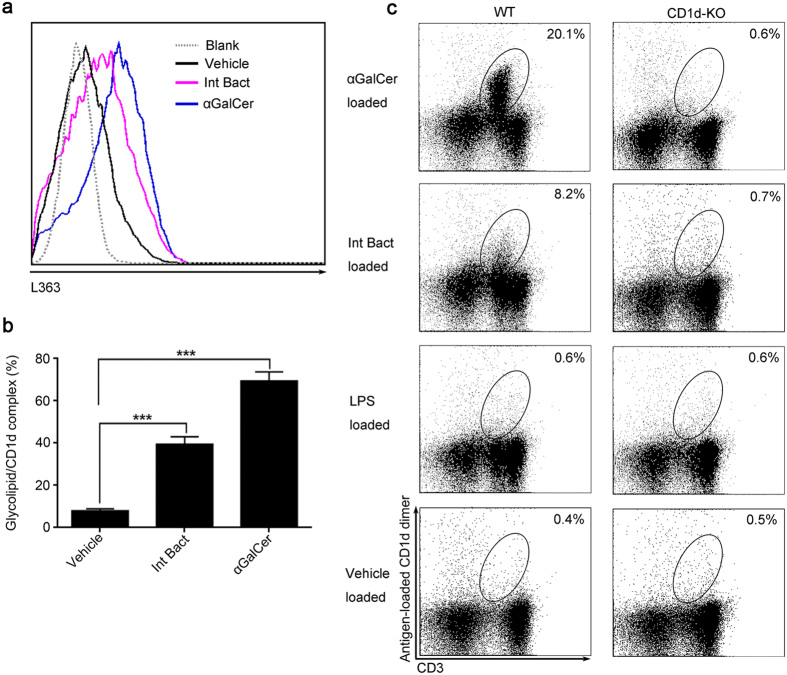
Intestinal bacteria contain NKT recognized glycolipids. (**a**,**b**) Sorted liver DCs from untreated SPF BALB/c mice were stimulated for 24 hr with αGalCer (200 ng), Int Bact or vehicle, respectively. Using L363, the presented glycolipids on CD1d were analyzed by flow cytometry, (**a**) the flow cytometric figure and (**b**) the statistical result were showed. (**c**) Staining of liver-infiltrating leukocytes from wild-type SPF C57BL/6 mice (WT) or CD1d-deficient mice with antigen-loaded CD1d dimer that constructed by loading CD1d:Ig protein with αGalCer, Int Bact, LPS or vehicle, respectively (see detail in methods). The data represent means ± SEM (n = 8, two independent experiments), ***P < 0.001, One-way ANOVA.

To examine whether the intestinal bacteria contains some antigen which can be served as NKT cell agonist, we cultured the hepatic DCs with αGalCer or Int Bact *in vitro*. Results showed that, similar to αGalCer, the glycolipid/CD1d complexes on DCs were also significantly increased in the Int Bact group (Fig. 7a,b). This result confirmed that intestinal bacteria contain αGalCer-like glycolipids, which can be presented by CD1d. And to determine whether NKT cells recognize the intestinal bacteria derived glycolipid antigens in the context of CD1d molecule, we stained the liver-infiltrating leukocytes with different antigen-loaded CD1d dimers^2^,^18^,^19^. CD1d dimer loaded with Int Bact bound to approximately 40% the number of cells that to αGalCer-loaded CD1d dimers (Fig. 7c). These data provided evidence for intestinal bacteria contain αGalCer-like glycolipids which can be presented by CD1d and recognized by NKT cells, suggesting that intestinal bacteria contain NKT cell agonist.

## Discussion

Gut microbiota have been implicated in many liver disorders, and NKT cells play a critical role in the progression of many liver diseases. However, the mechanistic link between gut bacteria and NKT cells in the development of liver disorders was remain unclear. This study showed that, in contrast to SPF mice, GF mice are protected from ConA-induced liver injury and that hepatic NKT cells from GF mice are resistant to activation following ConA treatment. We found that both CD1d-presented glycolipid antigens and circulating LPS levels are significantly increased in ConA-treated SPF mice compared with GF counterparts. However, we failed to detect differences in the functions of APC or NKT cells between SPF and GF mice. And heat-killed intestinal bacteria were able to restore ConA-induced NKT cell activation and liver injury in GF mice. Importantly, we also found intestinal bacteria contain αGalCer-like glycolipids which can be presented by CD1d and recognized by NKT cells. This study suggested that enterogenous bacterial glycolipids are important NKT cell agonist, and such antigens are presented by CD1d to activate NKT cells during ConA-induced hepatitis.

NKT cells play a central role in ConA-induced liver injury and activated NKT cells downregulate TCR and NK1.1 surface expression, which leads to an apparent reduction in NKT cell frequency^11^,^12^. Additionally, activated NKT cells would upregulate activation marker and produce large amounts of inflammatory cytokines^5^. In contrast to SPF mice, we found that ConA treated GF mice were resistant to contraction of the NKT cell population and the induction of IFN-γ. These findings indicated that hepatic NKT cells in GF mice are resistant to ConA-induced activation. We speculate that this might due to the absence of glycolipid antigens required for NKT cell activation, or a consequence of NKT cell and APC dysplasia in GF mice, and experiments were performed to find out the exactly mechanism.

In addition to αGalCer, a few studies have found that bacterial glycolipids can serve as ligands to activate NKT cells^1^,^2^. But whether intestinal bacteria contain NKT cell agonist, and the role of intestinal bacteria derived glycolipids in NKT cells mediated liver injury is not very clear. Using the L363 antibody, which can detect the CD1d-presented αGalCer or other glycolipids that are structurally similar to αGalCer, we found that the CD1d-presented glycolipids on hepatic leukocytes in ConA-treated GF mice were substantially lower compared with ConA-treated SPF mice. In addition, while less abundant in GF mice, CD1d-presented glycolipids could still be detected in these animals. It is believe that despite sterilization of the food, glycolipid antigens were still present and introduced into the GF animals. But the amount of such glycolipid antigens were relatively small when compared to SPF mice, due to SPF mice have normal intestinal bacteria. And such a small amount of glycolipids may be responsible for the generation, maturation or maintaining of hepatic NKT cells in GF mice. We also showed that LPS levels increased in the serum of SPF mice after ConA injection, but were undetectable in ConA-treated GF mice. These data confirmed that glycolipids were less abundant in GF mice, thus providing a possibly explanation for the failure of ConA to activate hepatic NKT cells in GF mice. A previous study showed that the translocation of enterogenous LPS into the peripheral blood was significantly increased in ConA-treated compared to control mice^20^. In both GF and SPF mice after ConA administration, we detected edema and intestinal villus degradation for reasons that remain unknown, and the levels of several cytokines, including IFN-γ, IL-4, G-CSF, RANTES, MIP-1a and IL-6 were increased in the intestine after ConA treatment. We also found ConA treatment significantly increased intestinal permeability to FITC-labeled dextran tracer. These suggested that ConA treatment can cause intestinal inflammation and increase intestinal permeability. It is reasonable that the translocation of bacterial glycolipids from the gut to the blood and liver increased after ConA injection in mice. Most importantly, we found that supplementation of some intestinal bacteria-derived glycolipids to GF mice prior to ConA injection could restore NKT cell activation-dependent liver injury, and results showed that these intestinal bacteria contain glycolipids which can be presented by CD1d and recognized by NKT cells. These findings confirmed that the absence of intestinal bacteria-derived glycolipids could impair NKT cell activation, which protects GF mice from ConA-induced liver injury.

In addition to absence of glycolipid antigen, the NKT cell and APC dysplasia might be another factor that contributes to the inability of NKT cells in GF mice. Park *et al*. showed that the phenotype, tissue distribution and function of NKT cells in C57BL/6 GF mice were similar to their SPF counterparts^21^. Olszak *et al*. found that the number of hepatic NKT cells were decreased in GF Swiss-Webster mice but increased in the GF mice of C57BL/6 genetic background; however the expression levels of several activation markers on NKT cells were unaltered in GF mice compared to their SPF counterparts^22^. But there are studies reported that NKT cells were reduced in GF C57BL/6 mice, and NKT cells from Swiss-Webster and C57BL/6 GF mice exhibited increased hyporesponsiveness to antigen stimulation than their SPF counterparts^23^,^24^. Collectively, these studies suggested that the number and functions of NKT cells in GF mice maybe change with the strains of the mice, the food they eat or the breeding environment. Additionally, few study detailed analyzed the development of hepatic NKT cells in BALB/c GF mice. In this study, we found that the αGalCer induced NKT-mediated liver injury is similar in SPF and GF mice. And *in vitro* experiment also confirmed that NKT cells from SPF and GF mice have similar function. These findings suggested that resistance of BALB/c GF mice to ConA-induced hepatitis is not due to NKT cell dysplasia.

The activation of NKT cells is closely linked to the presentation of glycolipid antigens by CD1d molecules, and studies have shown that αGalCer analogs can be presented most effectively by CD11c^+^DCs^16^. Walton *et al*. reported that the phenotype and *in vitro* antigen presenting functions of freshly isolated CD11c^+^DCs from spleens and mesenteric lymph nodes of BALB/c GF and SPF mice are very similar^25^. Consistent with this report, we found no difference in the frequency of liver CD11c^+^DC between PBS-treated GF and SPF mice. We further found no differences in the surface expression levels of CD1d, CD80, CD86 and MHC-II by liver CD11c^+^DC. Moreover, we showed that the αGalCer-presenting ability of DCs were similar between these two kind of mice *in vitro*. Based on the above, resistance of GF mice to ConA-induced liver injury was not due to immune cells dysplasia, but rather due to the relative absence of glycolipid antigens.

This study revealed that glycolipids derived from intestinal commensal organisms play an essential role in the activation of NKT cells and the liver injury induced following ConA treatment. However, which bacterial species are the predominant source of glycolipids for the activation of NKT cells remains to be determined. Previous studies suggested that *Sphingomonas*, *Borrelia burgdorferi*, *Streptococcus pneumoniae* and group B *Streptococcus* contain glycolipids that can be recognized by NKT cells^1^,^2^. Therefore, we speculated that the glycolipids in this present study may from the bacterial species which are structurally similar to these bacteria. Besides, this study used mAb L363 to visualize and quantify the glycolipids that presented by CD1d, L363 only recognize glycolipid/CD1d complexes formed by loading CD1d with αGalCer or other glycolipids which were structurally similar to αGalCer, such as C20:2 and OCH, and this mAb could not recognize CD1d without loading glycolipids^16^,^17^. Thus, we speculated that the glycolipids described in this present study were structurally similar to αGalCer. In addition, according to the manufacture’s protocol of Proimmune Company, NKT cells were identified as CD19^−^CD3^+^αGalCer/CD1d tetramer^+^ cells in this study. Although, there are many published studies regard CD19^−^CD3^+^αGalCer/CD1d tetramer^+^ cells as NKT cells^26^,^27^,^28^, it is more generally to identify NKT cells as TCRβ^+^αGalCer/CD1d tetramer^+^ cells. This created a limitation of this study, because different from TCRβ^+^ T cells, CD3^+^ T cells may also include some *γδ* T cells.

To address the relationship between NKT cell and intestinal bacterial glycolipids in liver disorders, we built liver injury models in SPF and GF mice. Our findings revealed that glycolipids from intestinal bacteria are important NKT cell agonist and are required during liver injury, and that the absence of bacterial glycolipids protects GF mice from NKT cell-associated liver injury progression. Our findings suggest that modulation of the intestinal microflora and intestinal glycolipids may represent a novel method for treating or preventing NKT cell-associated liver diseases.

## Materials and Methods

### Ethics statement

All methods of this study were carried out in accordance with the 1996 National Institutes of Health Guide for the Care and use of Laboratory Animals. And all experimental procedures were approved by the Animal Care Ethics Committee of the First Affiliated Hospital, Zhejiang University (Permit number: 2015-188).

### Mice

Six- to eight-week-old female mice were used in this study. BALB/c and C57BL/6 SPF mice were purchased from Shanghai SLAC Laboratory Animal Company. BALB/c GF mice were bred and maintained at the Third Military Medical University, Chongqing, China^29^,^30^. CD1d-deficient mice of C57BL/6 origin were purchased from the Jackson Laboratory (Bar Harbor, ME) and bred and maintained at University of Science and Technology of China, Hefei, China.

### ConA-induced hepatitis

Mice were injected intravenously through the tail vein with ConA (Vector Laboratories, Burlingame, CA). In general, the dose of ConA was 18 mg/kg and mice were sacrificed at 24 hr post-injection. But in survival analyses, mice were injected with 30 mg/kg ConA and observed for 72 hr. In some experiments, prior to ConA injection, we pretreated the GF mice with mixed intestinal bacteria or removed the intestinal bacteria of SPF by antibiotic treatment.

### Flow Cytometry

Anti-mouse αGalCer:CD1d complex antibody (L363, eBioscience, San Diego, CA)^17^ was used to detect glycolipid antigen presentation on CD1d. CD1d-tetramer loaded with αGalCer was purchased from ProImmune (ProImmune, Oxford, U.K.), unless otherwise specified, and NKT cells were identified as CD19^−^CD3^+^αGalCer/CD1d tetramer^+^ cells. Other antibodies used for staining were CD49b (HM ALPHA2), TCR-β (H57-597), CD11c (HL3), CD44 (IM7), CD80 (16-10A1), CD86 (GL1), IgG1 (A85-1), and I-Ad/I-Ed (2G9) were all purchased from BD Biosciences; CD3 (17A2), CD19 (6D5), TNF-α (MP6-XT22) and CD69 (H1.2F3) were from Biolegend; F4/80 (BM8), IFN-γ (XMG1.2), IgG2a (eBM2a) and IgG1 (eBRG1) were obtained from eBioscience. Data were acquired on a FACSCanto™ II flow cytometer (BD Biosciences) and analyzed with FlowJo (Treestar) or BD FACS Diva (BD Biosciences) software. The details of intracellular staining see the Supplementary Information.

### Cytokine and chemokine analysis

The cytokine and chemokine levels in the tissue homogenate and serum samples were generally assessed using the Luminex enzyme immunoassay (Luminex, TX, USA) according to the manufacturer’s protocol. In addition, IFN-γ and IL-4 concentrations in several experiments were detected by commercial ELISA kits (eBioscience). OPN level was detected by ELISA (R&D systems).

### Bacterial mixture

Heat-killed intestinal bacteria mixture (Int Bact) was prepared. The Int Bact was consisted of *E. coli, E. faecalis, Lactobacillus, Salmonella enteritidis* and *Group A Streptococcus* at equivalent amounts.

### Depletion of gut commensal microflora

Gut commensal microflora of SPF mice were removed by two methods as previously described^31^,^32^. Mice were treated with ampicillin (1 g/L; Sigma-Aldrich), neomycin sulfate (1 g/L; Sigma-Aldrich), vancomycin (500 mg/L; Shanghai Sangon Biotechnology, china) and metronidazole (1 g/L; Sigma-Aldrich) in their drinking water for four weeks^31^. Another method, mice were orally given neomycin sulfate (100 mg/kg) and streptomycin (100 mg/kg) twice per day for 7 days^32^.

### *In vitro* antigen-loaded CD1d-dimer assay

To determine whether NKT cells can recognize intestinal bacteria derived antigens, antigen-loaded CD1d:Ig protein staining cocktail were prepared according to the protocol of CD1d:Ig Recombinant Fusion Protein (Mouse DimerX; BD Biosciences)^2^,^18^,^19^. Briefly, we loaded CD1d:Ig protein with αGalCer, Int Bact, LPS or vehicle at 37 °C overnight, respectively. Cells were stained with these antigen-loaded CD1d:Ig protein staining cocktail for 60 min at 4 °C, followed by anti-mouseIgG1-PE (A85-1, BD Biosciences) and anti-CD3-APC antibody for 30 min at 4 °C^11^.

### Statistical analysis

Data are expressed as mean ± SEM and statistical analyses were performed using GraphPad Prism (GraphPad Software). Generally, differences between two groups were analyzed by Mann-Whitney U test, and multigroup comparisons were performed by One-way ANOVA. Comparisons of mortality were made by analyzing Kaplan-Meier survival curves, and the log-rank test was used to assess for differences in survival. P < 0.05 was considered significant. *P < 0.05, **P < 0.01, ***P < 0.001, ns represents not significant.

## Additional Information

**How to cite this article**: Wei, Y. *et al*. Enterogenous bacterial glycolipids are required for the generation of natural killer T cells mediated liver injury. *Sci. Rep*. **6**, 36365; doi: 10.1038/srep36365 (2016).

**Publisher’s note:** Springer Nature remains neutral with regard to jurisdictional claims in published maps and institutional affiliations.

## Supplementary Material

Supplementary Information
