# Supplementary material for: Enterogenous bacterial glycolipids are required for the generation of natural killer T cells mediated liver injury
Source: Sci Rep. 2016 Nov 8;6:36365. doi: 10.1038/srep36365 (PMC5099575; doi:10.1038/srep36365)
Supplement: Supplementary Information [file srep36365-s1.pdf]

Enterogenous bacterial glycolipids are required for the generation of natural killer T cells mediated liver injury

Yingfeng Wei[1,] , Benhua Zeng[2,] , Jianing Chen[1,] , Guangying Cui[1], Chong Lu[1], Wei Wu[1], Jiezuan Yang[1], Hong Wei[2,]*, Rufeng Xue[3, 4], Li Bai[3, 4], Zhi Chen[1], Lanjuan Li[1], Kazuya Iwabuchi[5], Toshimitsu Uede[6], Luc Van Kaer[7], and Hongyan Diao[1,] *

[1]State Key Laboratory for Diagnosis and Treatment of Infectious Diseases, Collaborative Innovation Center for Diagnosis and Treatment of Infectious Diseases, The First Affiliated Hospital, College of Medicine, Zhejiang University, Hangzhou, Zhejiang, 310003,China

[2]Department of Laboratory Animal Science, College of Basic Medical Sciences, Third Military Medical University, Chongqing, 400038, China

[3]Institute of Immunology and Key Laboratory of Innate Immunity and Chronic Disease of CAS, School of Life Sciences and Medical Center, University of Science and Technology of China, Hefei 230027, China

[4]Innovation Center for Cell Biology, Hefei National Laboratory for Physical Sciences at Microscale, Hefei 230027, China

[5]Department of Immunology, Kitasato University School of Medicine, Sagamihar, 108-8641, Japan

[6]Molecular Immunology, Institute for Genetic Medicine, Hokkaido University, Sapporo, 0600815, Japan

[7]Department of Microbiology and Immunology, Vanderbilt University School of Medicine, Nashville, Tennessee, 37232

Supplementary figures

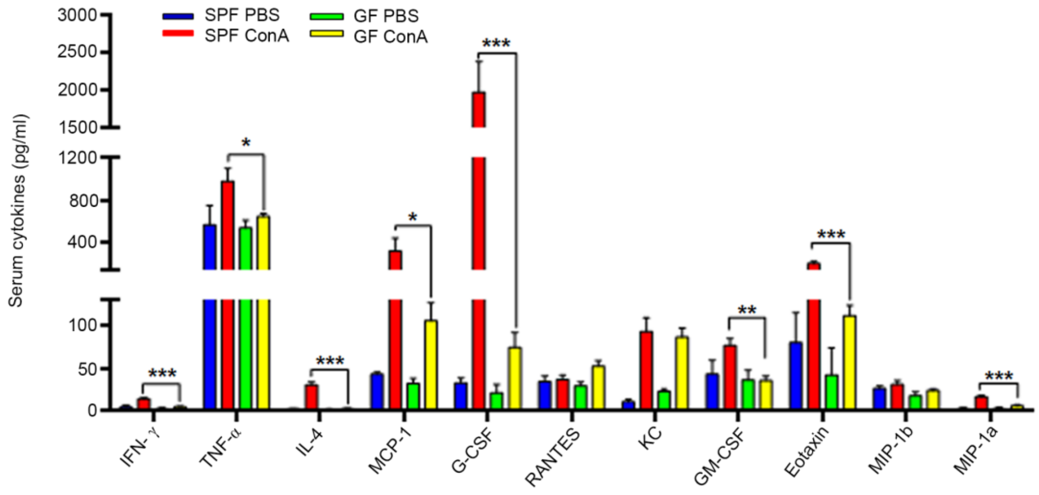

Supplementary Fig. S1. Reduced serum cytokine amount in ConA treated GF mice. SPF and GF BALB/c mice were treated with ConA or PBS, sacrificed 24 hr later. Cytokine profile in serum was analyzed by the Luminex enzyme immunoassay system. The data represent means ± SEM (n=8, two independent experiments), *P < 0.05, **P < 0.01, ***P < 0.001, One-way ANOVA.

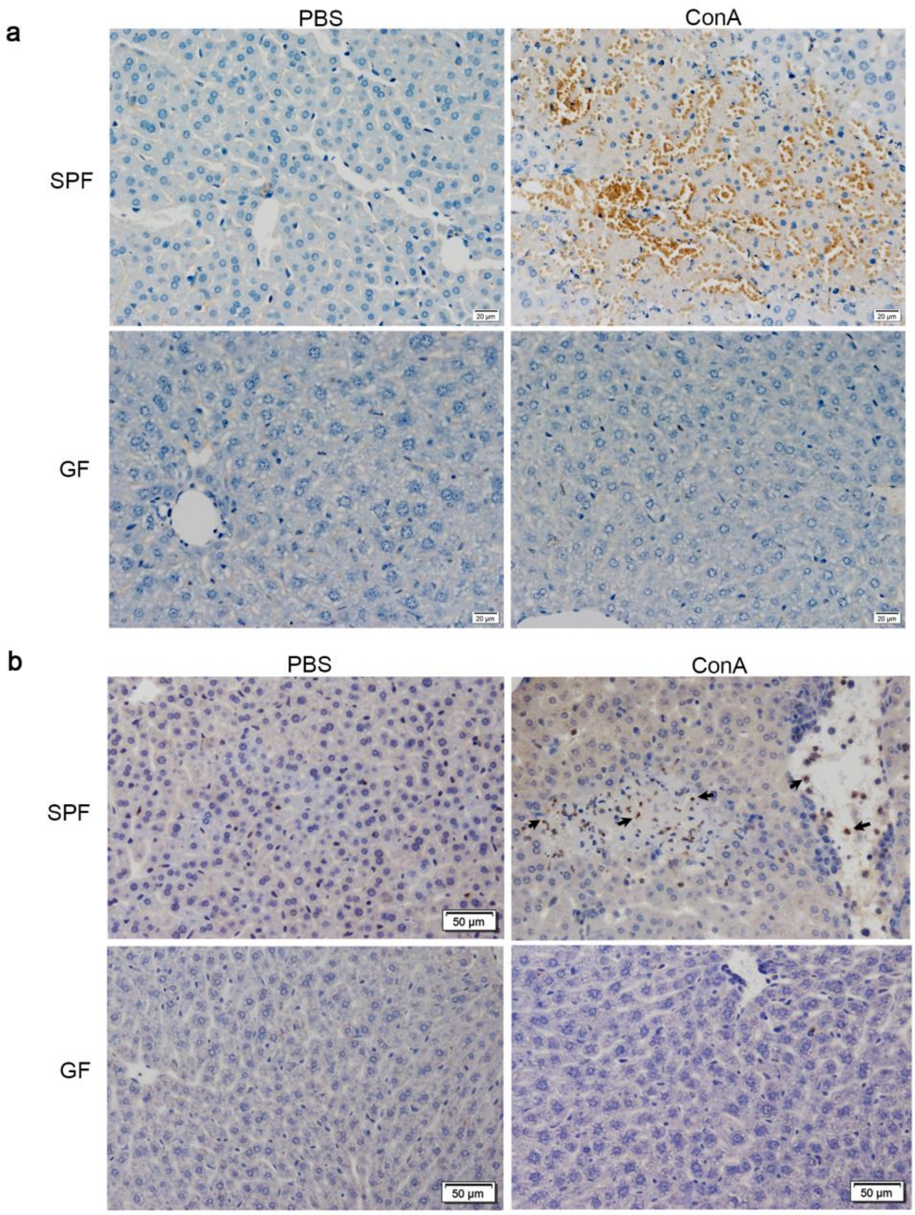

Supplementary Fig. S2. Reduced neutrophil cells infiltration and activation in ConA treated GF mice. BALB/c mice were treated with ConA or PBS, sacrificed 24 hr later. **(a)** Immumohistochemical staining of neutrophil cells in the liver sections, detected by Gr-1, original magnification, 400×. Scale bars represents 20 μm. **(b)** Hepatic myeloperoxidase (MPO) was analyzed by immumohistochemical staining, original magnification, 200×, scale bars represents 50 μm. Two independent experiments with similar results were performed.

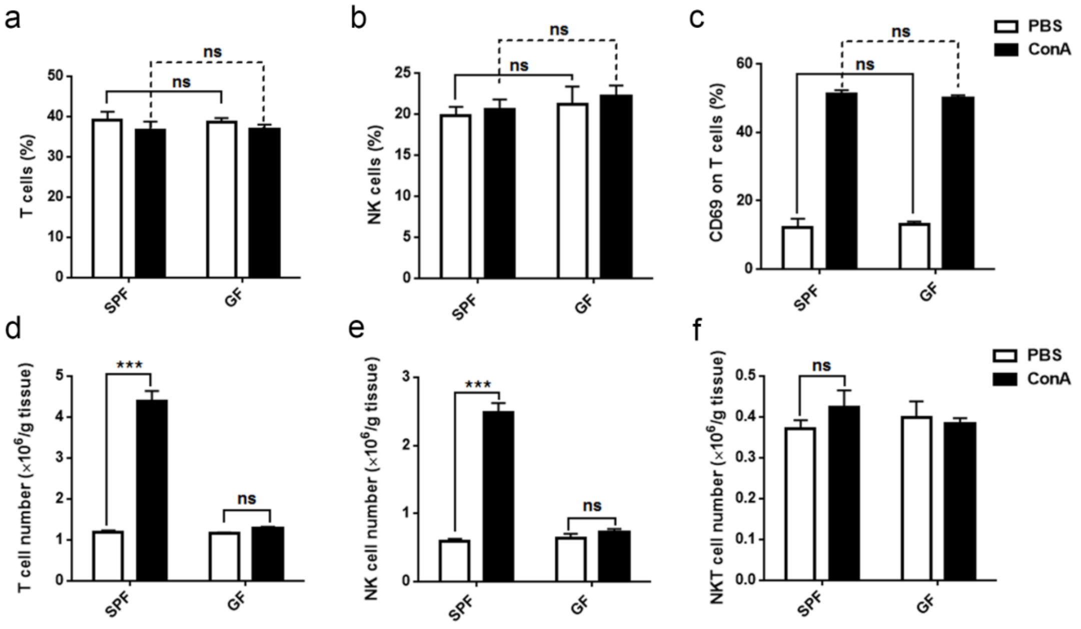

Supplementary Fig. S3. Measurements of hepatic conventional T cells and NK cells. The percentages of hepatic **(a)** conventional T cells, **(b)** NK cells, **(c)** the surface CD69 expression of hepatic conventional T cells. The absolute number of hepatic **(d)** conventional T cells, **(e)** NK cells, **(f)** and NKT cells. Data were acquired from the mice intrahepatic leukocytes 24 hr after PBS or ConA injection, analyzed by flow cytometry. The data represent means ± SEM (n=6, two independent experiments), ns represents not significant, ***P < 0.001, One-way ANOVA.

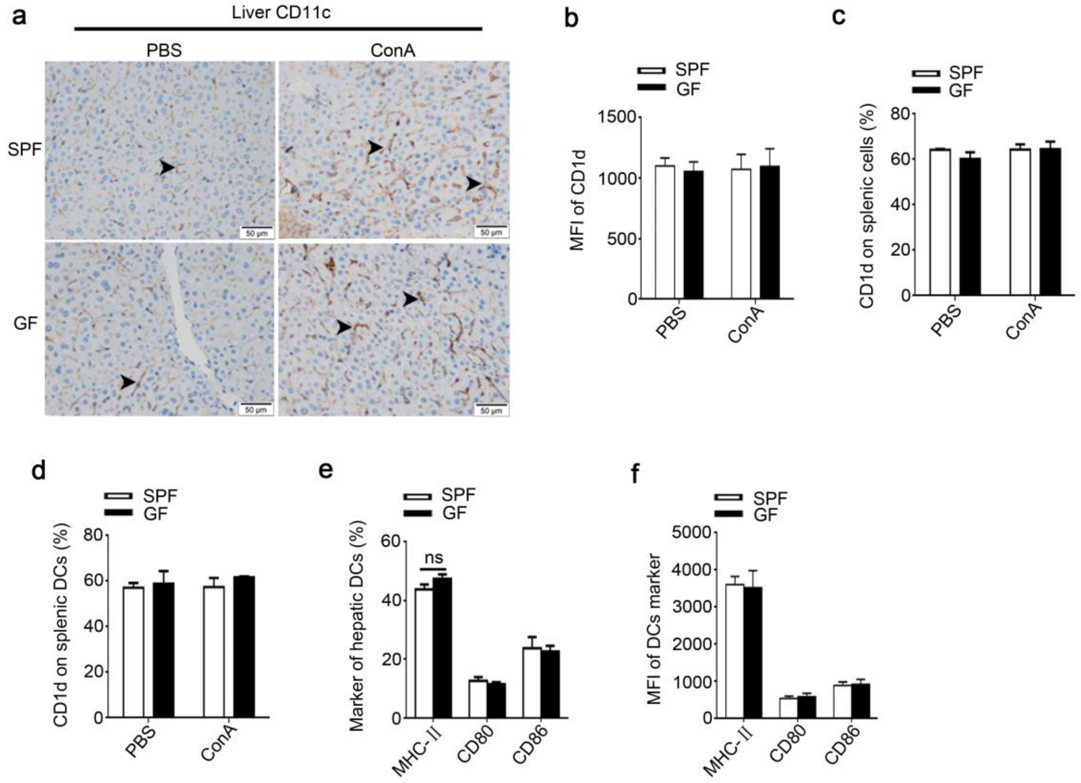

Supplementary Fig. S4. Measurements of DCs and CD1d. **(a-d)** Samples were obtained at 24 hr after ConA or PBS treatment to SPF and GF BALB/c mice. **(a)** Immunohistochemical detection of CD11c in the liver sections, original magnification, 200×. Scale bars represent 50 μm. **(b)** MFI levels of CD1d on hepatic DCs. The percentage of CD1d on **(c)** total splenic cells and **(d)** splenic DCs were analyzed by flow cytometry. **(e-f)** Samples were obtained from untreated mice (without PBS or ConA injection). **(e)** Expression of MHC–II, CD80 and CD86 on hepatic CD11c^{+} DCs, and **(f)** the MFI levels of these markers on hepatic DCs, analyzed by flow cytometry. The data represent means ± SEM (n=8, two independent experiments), ns represents not significant, One-way ANOVA.

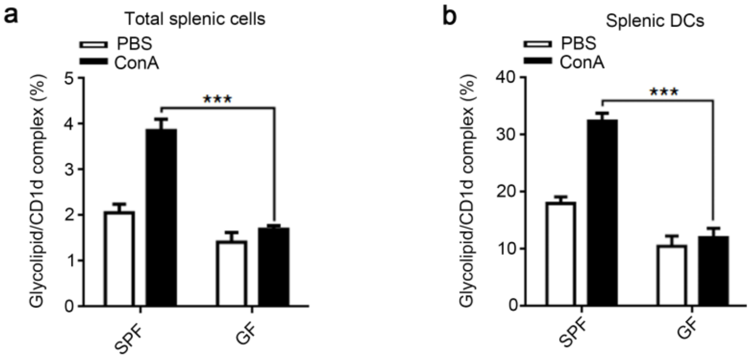

Supplementary Fig. S5. The expression of Glycolipid/CD1d complex in splenic cells. Cells were isolated from the spleen 24 hr after PBS or ConA treatment. Glycolipid/CD1d complex expression on total splenic cells **(a)** or splenic DCs **(b)** was analyzed by flow cytometry. The data represent means ± SEM (n=8, two independent experiments), ***P < 0.001, One-way ANOVA.

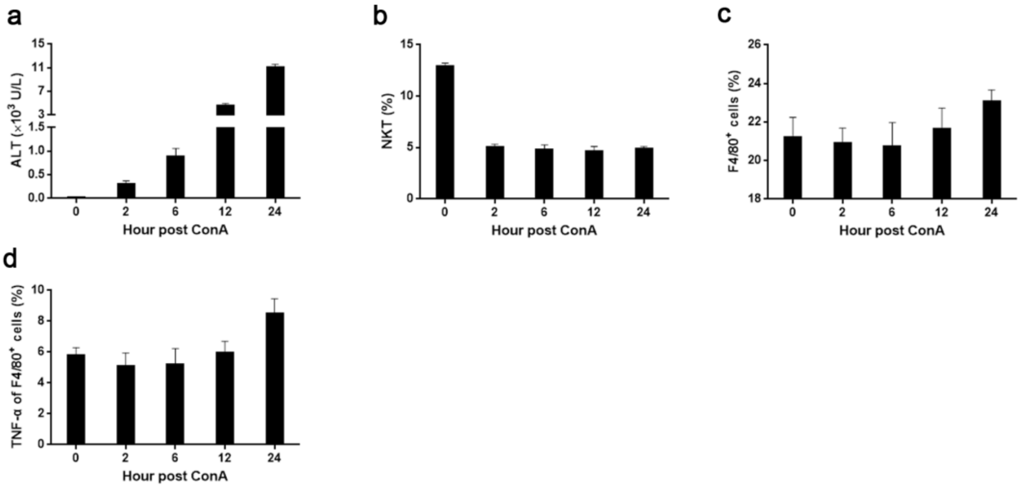

Supplementary Fig. S6. Kupffer cell is less important than NKT cell in ConA hepatitis. We analyzed the **(a)** ALT level, **(b)** hepatic NKT cell percentage, **(c)** the hepatic F4/80$^+$ cell percentage and **(d)** intracellular TNF-α level of hepatic F4/80$^+$ cell. Data were obtained by flow cytometry from the SPF BALB/c mice at 0hr, 2hr, 6hr 12hr, and 24hr after ConA injection (18mg/kg). The data represent means ± SEM.

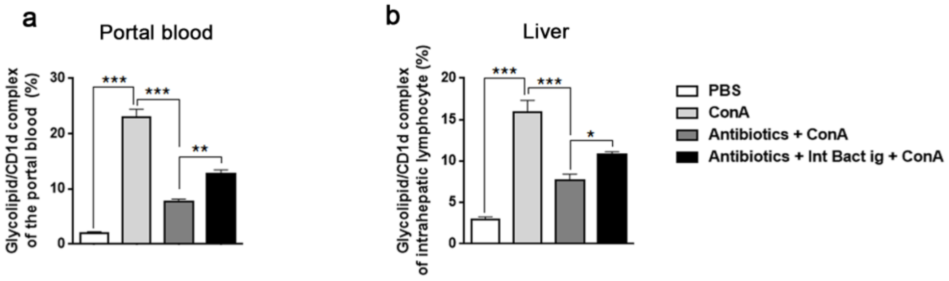

Supplementary Fig. S7. Increased glycolipids presenting by CD1d were from intestinal microbiota. BALB/c SPF mice were divided into four groups. In PBS group, mice were sacrificed 24 hr after a PBS injection. In ConA group, mice were sacrificed 24 hr after ConA (18mg/kg) injection. In Antibiotics + ConA group, to remove most of the intestinal bacteria, mice received gavage with neomycin sulfate (100 mg/kg) and streptomycin (100 mg/kg) twice per day for 7 days, then these mice were injected with ConA (18mg/kg) and sacrificed 24 hr later. In Antibiotics + Int Bact ig + ConA group, mice were orally given neomycin sulfate (100 mg/kg) and streptomycin (100 mg/kg) twice per day for 7 days, then these mice received 500 µl Int Bact (2×109 CFU/ml) by intragastric gavage (ig) daily for 5 days, and then these mice were injected with ConA (18mg/kg) and sacrificed 24 hr later. Int Bact was heat-killed intestinal bacteria mixture which consisted of E. coli, E. faecalis, Lactobacillus, Salmonella enteritidis and Group A Streptococcus. The percentage of glycolipid/CD1d complex in both **(a)** portal blood lymphocyte and **(b)** the intrahepatic leukocytes were analyzed. The data represent means ± SEM (n=6, two independent experiments), *P < 0.05, **P < 0.01, ***P < 0.001, One-way ANOVA.

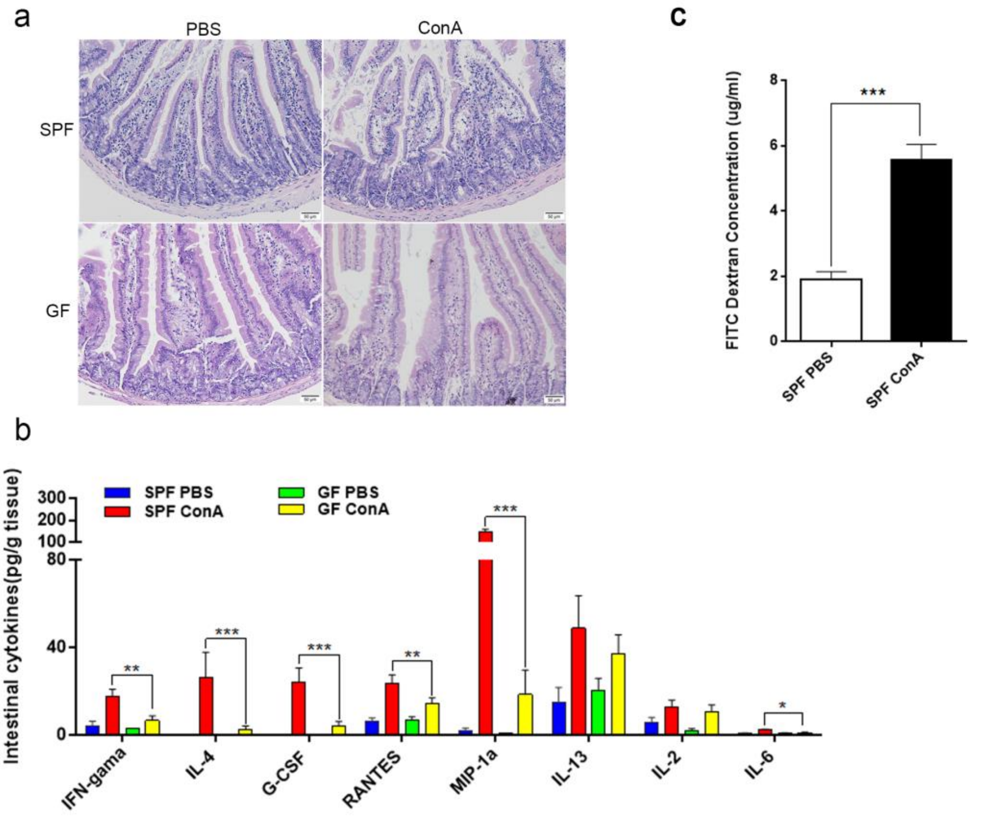

Supplementary Fig. S8. ConA causes intestinal inflammation and increases intestinal permeability. Mice were sacrificed 24 hr after Con A (18 mg/kg) or PBS treatment. **(a)** H&E staining of small intestine; original magnification, 200×. Scale bars=50μm. **(b)** Cytokine profile of the small intestine. **(c)** Four hour prior to sacrifice, mice were gavaged with 60 mg/100 g body weight of FITC-dextran, serum FITC-dextran was quantified as a measure of intestinal permeability. The data represent means ± SEM (n=6, two independent experiments), *P < 0.05, **P < 0.01, ***P < 0.001, One-way ANOVA.

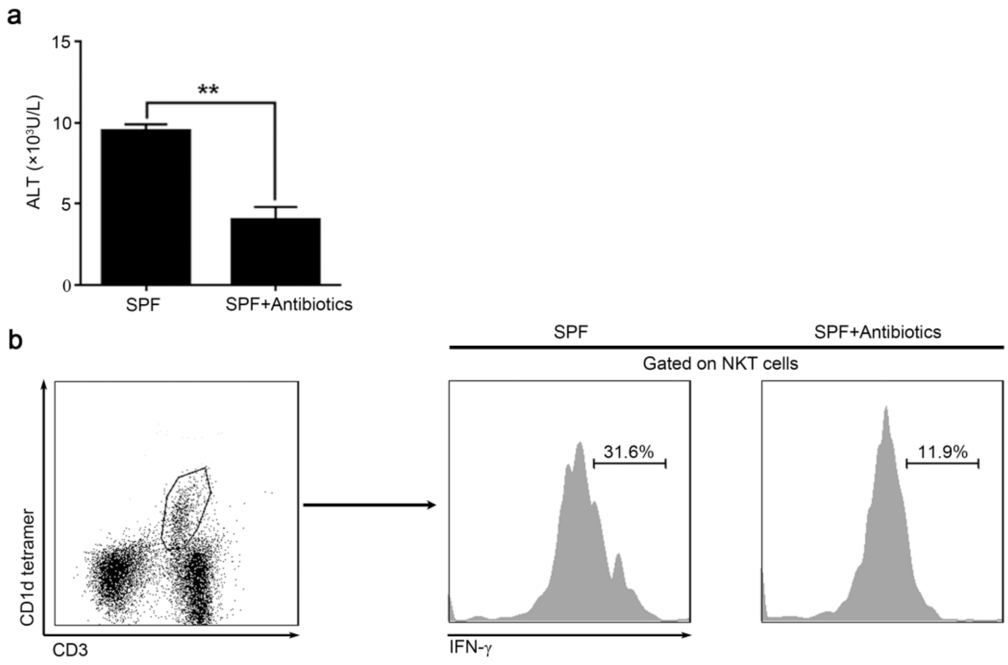

Supplementary Fig. S9. Antibiotics administration ameliorated ConA-induced liver injury. SPF BALB/c mice were removed intestinal bacteria by a cocktail of antibiotic in their drinking water for four weeks. **(a)** The ALT level in the serum and **(b)** the intracellular levels of IFN–γ in the hepatic NKT cells ($CD19^{-}CD3^{+}$αGalCer/CD1d tetramer^{+}) were analyzed 24 hr after ConA injection. The data represent means ±SEM (n=8, two independent experiments), **$P < 0.01$, Mann-Whitney U test.

Supplementary methods

Animal treatment and reagent. At the indicated times, ConA- or αGalCer-treated and control mice were sacrificed. The ALT and AST levels in serum samples were detected using a standard clinical automated analyzer (SRL, Tokyo, Japan). KRN7000 (αGalCer) was purchased from Funakoshi Company and dissolved according to the manufacturer's protocols. Cells (2×10^6/ml) were cultured in RPMI-1640 (Gibico) with 10% FBS (Gibico).

Bacterial mixtures. Heat-killed intestinal bacteria mixture (Int Bact) was prepared for this study. Int Bact was consisted of *E. coli*, *E. faecalis*, *Lactobacillus*, *Salmonella enteritidis* and Group A *Streptococcus* at equivalent amounts. The concentrations of Int Bact used for intragastric gavage (ig) and intravenous injection (iv) were 2×10^9 colony-forming units (CFU)/ml and 2.5×10^8 CFU/ml, respectively. After mixing, the bacteria were heat-killed by 2hr exposure to 74℃ as described elsewhere [1]. In addition, *E. coli*, *E. faecalis*, and *Lactobacillus* were all isolated from human feces by our laboratory. Group A *Streptococcus* and *Salmonella enteritidis* were isolated from humans and maintained in our laboratory as previously described[2,3].

ConA-induced hepatitis. In some experiments, were moved the intestinal bacteria of SPF by antibiotic treatment or pretreated the GF mice with mixtures of heat-killed intestinal bacteria by intragastric gavage (ig) or intravenous injection (iv) prior to

ConA injection. In the experiments of pretreating GF mice with mixed intestinal bacteria, 6-week-old GF mice were divided into 4 groups. In group 1 (ConA + PBS), mice received 500 µl PBS ig daily for 8 days, and ConA was injected immediately after the last orally administration PBS . In group 2 (PBS + Int Bact ig), mice received 500 µl Int Bact (2×10^9 CFU/ml) by ig daily for 8 days, and PBS was injected immediately after the last orally administration Int Bact. In group 3 (ConA + Int Bact ig), mice received 500 µl Int Bact (2×10^9 CFU/ml) by ig daily for 8 days, and ConA was injected immediately after the last orally administration Int Bact. In group 4 (ConA + Int Bact iv), we did not administer any treatment for the first 7 days, and injected 200 µl of Int Bact (2.5×10^8 CFU/ml) on the 8th day via the tail vein, and 1 hr later ConA was injected. At 24 hr after ConA injection, all mice in the 4 groups were sacrificed and analyzed.

Cell preparations. To isolate intrahepatic leukocytes, we used a protocol that was previously described [4]. Briefly, livers were minced and then pressed through a mesh filter. After washing, cells were suspended in 33% Percoll solution (GE Healthcare) and centrifuged at 2000 rpm for 15 min. The resuspended parenchymal cells were discarded and the pellets were treated with red blood cell (RBC) lysis solution. Leukocytes were resuspended in DMEM with 10% FCS for subsequent analysis. Additionally, splenocyte suspensions were prepared by grinding spleens through mesh filters, and then treated with RBC lysis solution.

Intracellular staining. We detected the intracellular IFN-γ of NKT ($CD19^{-}CD3^{+}$αGalCer/CD1d tetramer^{+}) *in vivo* and *in vitro*. *In vivo*, the freshly isolated intrahepatic leukocytes were performed as the procedures of intracellular IFN-γ detection without any additional stimulation. *In vitro*, splenocytes (2×10^{6}/ml) were stimulated for 24hr with αGalCer (200 ng/ml) or vehicle before perform the procedures of intracellular IFN-γ detection. For intracellular staining, cells were stimulated for 4 hr with PMA (50 ng/ml) and ionomycin (250 ng/ml; both from Sigma-Aldrich) in the presence of GolgiPlug (BD Biosciences), after surface staining, fixed and made permeable according to the manufacturer's instructions (BD Biosciences). Data were acquired on a FACSCanto™ II flow cytometer (BD Biosciences) and analyzed with FlowJo (Treestar) or BD FACS Diva (BD Biosciences) software.

Cell sorting. Intrahepatic leukocytes were obtained from untreated C57BL/6 SPF mice DC cells were sorted as $CD11c^{+}$ on a FACS Aria III instrument (Becton Dickinson). The purity of the sorted cells was > 95% as verified by post-sort analysis.

Histological analyses. To analyze morphological changes, tissue samples were paraffin-embedded, sectioned and stained with hematoxylin and eosin (H&E). The primary antibodies used for immunohistochemistry were F4/80 (sc-377009, Santa Cruz), CD11c (N418, Abcam), MPO (ab134132, Abcam) and Gr1 (ab25377, Abcam). Additionally, liver apoptosis was measured by terminal deoxynucleotidyl

transferase-mediated dUTP nick end labeling (TUNEL) staining of paraffin-embedded slides (Calbiochem, La Jolla, CA).

Endotoxin Assay. Under chloral hydrate anesthesia, blood was isolated from the portal vein during laparotomy in pyrogen-free heparinized syringes. Plasma endotoxin (LPS) was measured using the Limulus amebocyte lysate pyrogen test (Xiamen Houshiji, Ltd., Xiamen, China). Levels of endotoxin in plasma from GF mice were all below the limit of detection.

Intestinal Permeability In Vivo. Mice were injected intravenously through the tail vein with ConA (18 mg/kg) or PBS, and sacrificed at 24 hr post-injection. As described previously[5-7], 4 hr prior to sacrifice, mice were gavaged with 60 mg/100 g body weight of FITC-dextran (mol wt 4,000; Sigma Aldrich). Cardiac puncture was performed and serum analysis of FITC concentration performed.

Supplementary references

1 Mattner, J. *et al.* Exogenous and endogenous glycolipid antigens activate NKT cells during microbial infections. *Nature* **434**, 525-529 (2005).

2 Diao, H. & Kohanawa, M. Endogenous interleukin-6 plays a crucial protective role in streptococcal toxic shock syndrome via suppression of tumor necrosis factor alpha production. *Infection and immunity* **73**, 3745-3748, doi:10.1128/iai.73.6.3745-3748.2005 (2005).

3 Li, Y. T. *et al.* Effects of gut microflora on hepatic damage after acute liver injury in rats. *The Journal of trauma* **68**, 76-83, doi:10.1097/TA.0b013e31818ba467 (2010).

4 Diao, H. *et al.* Osteopontin as a mediator of NKT cell function in T cell-mediated liver diseases. *Immunity* **21**, 539-550, doi:10.1016/j.immuni.2004.08.012 (2004).

5 Napolitano, L. M., Koruda, M. J., Meyer, A. A. & Baker, C. C. The impact of femur fracture with associated soft tissue injury on immune function and intestinal permeability. *Shock (Augusta, Ga.)* **5**, 202-207 (1996).

6 Synnestvedt, K. *et al.* Ecto-5'-nucleotidase (CD73) regulation by hypoxia-inducible factor-1 mediates permeability changes in intestinal epithelia. *The Journal of clinical investigation* **110**, 993-1002, doi:10.1172/jci15337 (2002).

7 Furuta, G. T. *et al.* Hypoxia-inducible factor 1-dependent induction of intestinal trefoil factor protects barrier function during hypoxia. *The Journal of*

experimental medicine **193**, 1027-1034 (2001).
